# Magnetic resonance arthrographic demonstration of knee joint communication in isolated popliteal artery cystic adventitial disease

**DOI:** 10.1016/j.jvscit.2026.102453

**Published:** 2026-08-14

**Authors:** Nontaphon Piyawattanametha, Rahul Gogoi, Tomas Marek, Manju Kalra, Kimberly K. Amrami, Robert J. Spinner

**Affiliations:** a Department of Neurosurgery, Mayo Clinic, Rochester, MN; b Department of Radiology, Mayo Clinic, Rochester, MN; c Division of Vascular and Endovascular Surgery, Mayo Clinic, Rochester, MN

**Keywords:** Cystic adventitial disease, Popliteal artery, MR arthrography, Articular theory, Joint communication

## Abstract

Cystic adventitial disease is a rare, nonatherosclerotic condition most commonly affecting the popliteal artery in the popliteal fossa. We describe the case of a 44-year-old man with episodic calf claudication in whom duplex ultrasound and computed tomography angiography showed cystic lesions surrounding the popliteal artery with mild luminal narrowing, consistent with cystic adventitial disease. Conventional magnetic resonance (MR) imaging identified a popliteal artery adventitial cyst with suspected knee joint connection. Fluoroscopically guided intra-articular gadolinium injection in the knee followed by 3-T MR arthrography confirmed cyst communication with the knee joint. This case supports the articular theory and highlights MR arthrography as a problem-solving tool in selected cases.

Cystic adventitial disease (CAD) is a rare, nonatherosclerotic condition characterized by mucinous cystic fluid within the adventitia of a blood vessel, most frequently the popliteal artery.^1^ Cyst expansion can compress the vessel lumen and cause intermittent claudication, often in patients without typical atherosclerotic risk factors. Although standard vascular imaging can establish the diagnosis by showing a cystic lesion related to the vessel wall, understanding the mechanism requires identifying cyst communication with the adjacent joint.^1^^,^^2^ Identifying this pathway may inform targeted treatment.

Several theories have been proposed since Atkins and Key’s 1947 description. We support the articular, or synovial, theory in all cases, although joint connections have been identified in only 122 of 729 reported cysts (17%).^1^^,^^3^ This theory suggests that CAD develops when synovial fluid escapes from an adjacent joint and tracks along an articular vascular branch into the parent vessel adventitia. For popliteal artery CAD, the middle genicular artery likely acts as the conduit.^1^

Magnetic resonance imaging (MRI) can identify a joint connection by depicting the cyst-joint tract, whereas arthrography can confirm communication by demonstrating intra-articular contrast passage into the cyst. Previous imaging and arthrographic observations have supported joint connections in CAD, but MR arthrographic confirmation of communication through this pathway in isolated CAD remains lacking.^4^, ^5^, ^6^, ^7^

We describe the case of isolated popliteal artery CAD in which 3-T MR arthrography confirmed communication between the knee joint and the adventitial cyst. Written patient consent was obtained for publication.

## Report

A 44-year-old man, a healthy nonsmoking construction worker, presented with a 9-month history of intermittent left calf claudication. Symptoms were episodic, occurring in four to five discrete 1-month episodes. During symptomatic periods, claudication developed after walking approximately 30 m and was relieved by rest. He remained physically active between episodes. His symptoms arose spontaneously, with no documented knee trauma, vascular injury, arthritis, or relevant family history.

Both lower limbs were warm, with palpable pulses. Neurological examination was normal, without Tinel sign or popliteal tenderness.

**Fig 1:**
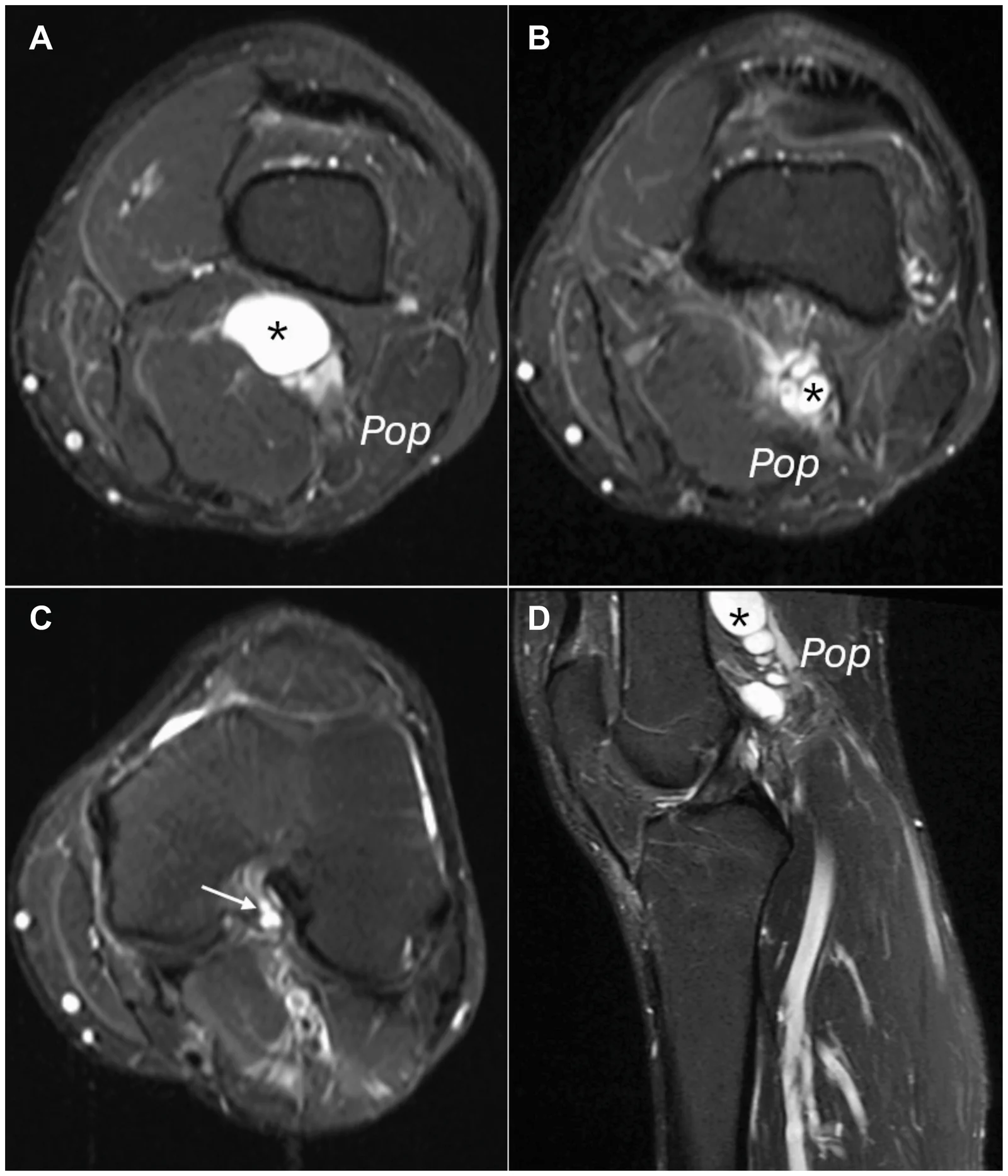
Initial outside 1.5-T magnetic resonance (MR) examination of the left leg using short tau inversion recovery (STIR) sequences. **A-C,** Axial images show a multilobulated cyst (∗) in the popliteal fossa surrounding the popliteal vessels. In **(C)**, the *white arrow* indicates a possible communication between the cyst and the knee joint. **D,** Sagittal image shows the cyst (∗) posterior to the distal femur. *∗*, Cyst; *Pop*, popliteal vessels.

Electromyography was normal. Physiologic testing showed normal bilateral Doppler waveforms, normal resting ankle-brachial index values (right posterior tibial/dorsalis pedis, 1.11/1.04; left, 1.08/1.07), and normal postexercise ankle-brachial index values after 15 minutes at 4 mph/5% grade without symptoms (right, 1.19; left, 0.98). Dorsiflexion/plantarflexion maneuvers for popliteal artery entrapment were negative. Duplex ultrasound examination showed patent left femoral, popliteal, posterior tibial, and dorsalis pedis arteries without significant stenosis and multiloculated cysts abutting the popliteal artery (1.3 × 1.8 × 6.9 cm). Computed tomography (CT) angiography showed multiloculated cysts surrounding the popliteal artery (2.4 × 2.0 × 3.1 cm), with mild narrowing and patent tibioperoneal arteries. An outside 1.5-T lower-leg MR study obtained 5 months earlier incompletely visualized this region and suggested possible cyst-knee joint communication (Fig 1, *A-D*), prompting dedicated 3-T MR arthrography.

**Fig 2:**
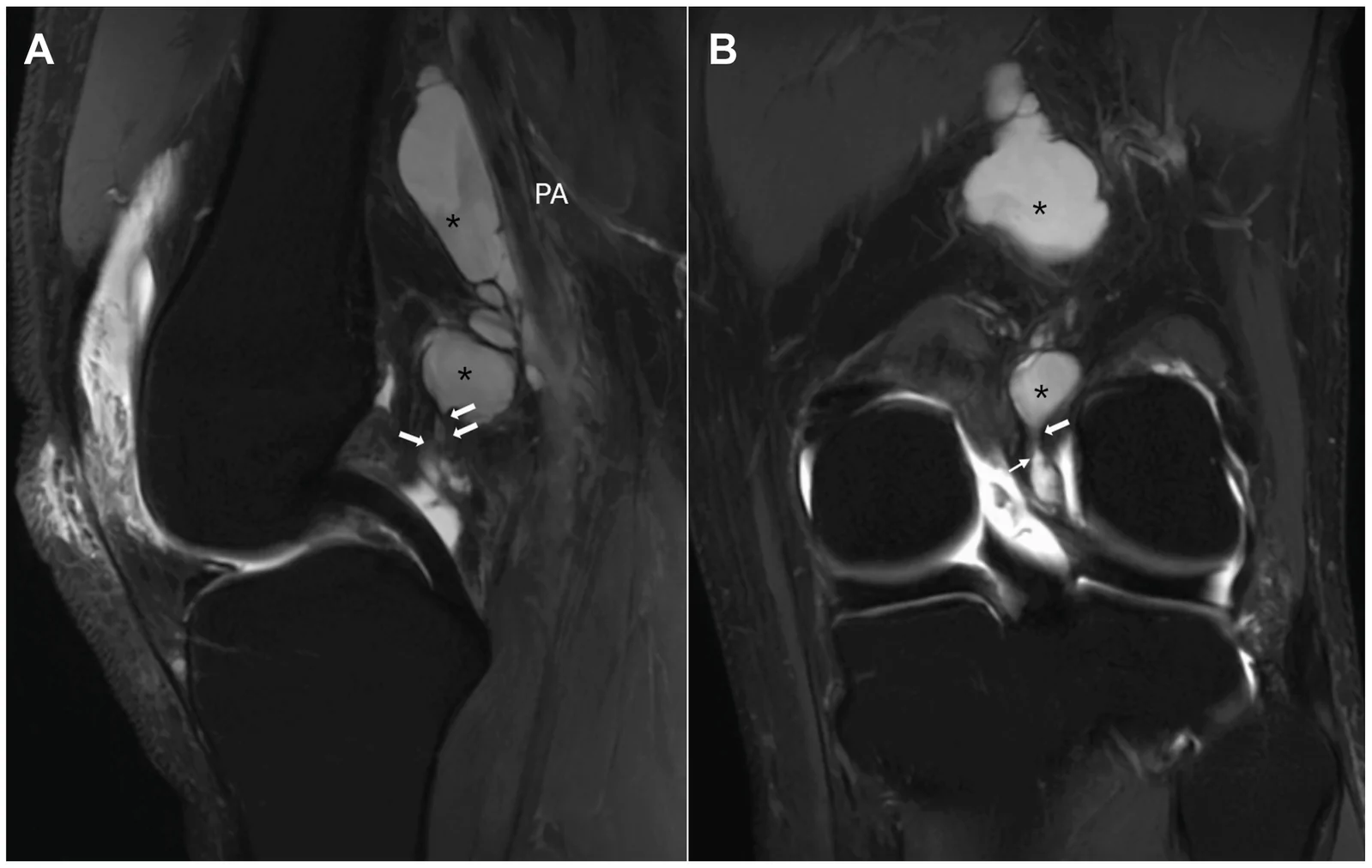
Multiplanar fat-suppressed T2-weighted magnetic resonance (MR) arthrographic images of the left popliteal fossa. **A,** Sagittal image shows the cyst neck (*bold arrow*). A fluid-fluid level is present within the uppermost cyst component, consistent with gravitational layering of intracystic fluid of differing composition. **B,** Coronal image shows the cyst neck and its connection to the knee joint (*white arrow*). *∗*, Cyst; *PA*, popliteal artery.

**Fig 3:**
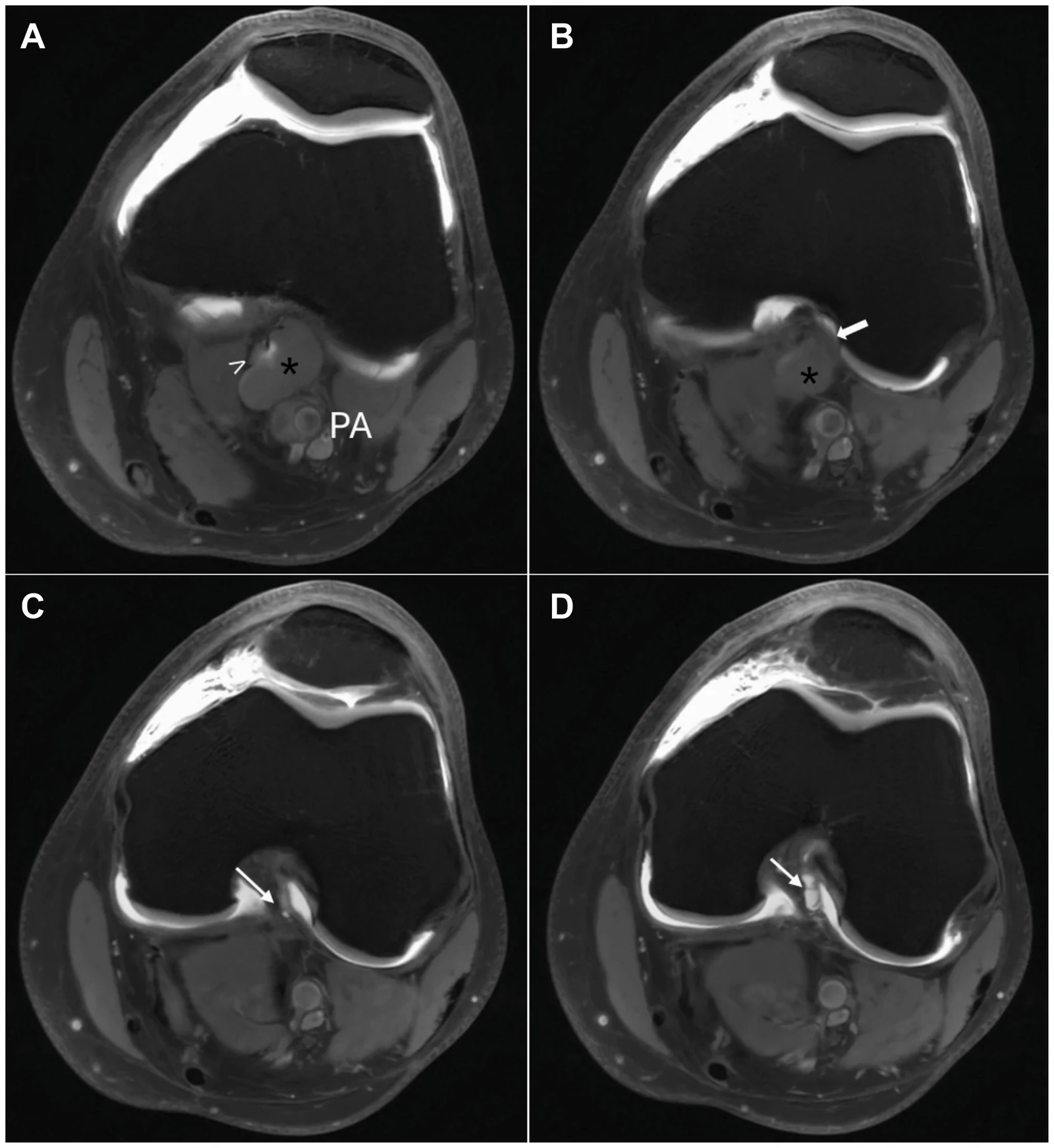
Delayed axial fat-suppressed T1-weighted magnetic resonance (MR) arthrographic images of the left popliteal fossa obtained after knee flexion and extension. **A,** The adventitial cyst (∗) surrounds the popliteal artery (*PA*), with arthrographic contrast within the cyst (*arrowhead*), confirming communication with the knee joint. **B,** The communicating neck (*bold arrow*) connects the cyst to the posterior joint capsule. **C** and **D,** Consecutive images show the joint-cyst communication (*white arrows*). *∗*, Cyst.

After fluoroscopic confirmation of intra-articular position with iodinated contrast, dilute gadolinium was injected and 3-T MR arthrography was obtained. Sagittal and coronal fat-suppressed T2-weighted images demonstrated a thin elongated neck arising from the posterior joint capsule adjacent to the posterior cruciate ligament and extending toward the adventitial cyst (Fig 2, *A* and *B*). Delayed axial fat-suppressed T1-weighted imaging after a short interval of knee flexion/extension showed increased intracystic signal, confirming joint communication (Fig 3, *A-D*). The examination also showed a medial meniscal tear and focal 2-mm lateral tibial plateau chondral defect.

Symptoms resolved spontaneously and he remained asymptomatic while walking 3 miles daily. Nonoperative management with surveillance ultrasound was elected.

## Discussion

The central finding is not simply the presence of a popliteal artery adventitial cyst, but progression from an initial MR suggestion of a possible joint connection to MR arthrographic confirmation of communication between the knee joint and the cyst, as predicted by the articular theory. Intra-articular contrast extended from the knee through a thin capsular neck near the posterior cruciate ligament into the adventitial cyst, with increased intracystic T1 signal after a short interval of knee flexion and extension. This demonstrates the complete source-conduit-target pathway: synovial origin from the knee, propagation through an articular vascular branch, and extension into the parent popliteal artery adventitia.

Historically, joint connections were recognized in occasional cases of CAD before integration into a coherent mechanism. In 1961, Parkes described wrist-originating vascular examples in which dissecting ganglia within the adventitia had operative joint connections.^8^ Later, CAD reports used early plain arthrography, CT arthrography, and delayed postarthrographic imaging to visualize knee joint connections.^4^, ^5^, ^6^ To our knowledge, this is the first report of MR arthrographic confirmation of joint communication in isolated CAD.

These historical observations for CAD were later integrated into a broader conceptual framework derived from the unifying articular theory of intraneural ganglion cysts (IGCs), an analogous clinical entity in which mucinous cysts develop within the epineurium of peripheral nerves.^3^ In IGCs, the cyst itself is the terminal manifestation of a joint-derived process: synovial fluid exits the joint, travels along an articular neural branch, and propagates within the parent nerve.^3^ The same source-conduit-target model applies to adventitial cysts, with an articular vascular branch replacing the articular neural branch.^2^ This analogy reframes CAD not as a primary vessel wall disorder, but as a joint-derived cystic process with vascular expression. MR arthrography has previously supported this mechanism in combined intraneural and adventitial cysts^7^; this case extends that logic to isolated popliteal artery CAD without an associated intraneural cyst.

In routine vascular evaluation, duplex ultrasound examination and CT angiography characterize flow disturbance, luminal narrowing, and regional anatomy, but may not reliably demonstrate the small articular branch linking the cyst to the joint. This is where cross-specialty translation is useful. Imaging principles developed for IGCs can be applied directly to CAD: rather than focusing only on the terminal cyst, radiologists should look for the joint of origin and the articular branch that connects it to the parent structure. This principle is reinforced by a radial artery CAD series in which all cases had identifiable joint connections on MRI when specifically evaluated.^9^ MRI, and in selected cases MR arthrography, may define the anatomical route by which the cyst reaches the vessel, beyond what ultrasound examination or CT angiography typically show.^1^^,^^2^^,^^7^

This distinction may explain why recurrence and persistence remain difficult to interpret in CAD. Many reports have limited follow-up and no routine postoperative imaging, so subclinical persistence may be missed. Procedures directed only at the cyst or the vessel, whether lumen or adventitia, may leave the articular conduit intact, providing a plausible explanation for recurrence after cyst- or vessel-directed treatment. Identifying the connection preoperatively may shift management toward mechanism-based treatment, in which the articular branch is sought and disconnected when intervention is required.^1^^,^^2^

This report is limited by its single-patient design and lack of operative confirmation. As the patient was managed nonoperatively, this case cannot determine whether MR arthrographic identification of the conduit changes outcomes. Larger studies with operative correlation and longer imaging follow-up are needed to determine how often MR arthrography identifies these communications and whether targeting the articular branch reduces clinical or subclinical recurrence.

## Conclusions

MR arthrography can confirm and anatomically define joint communication in selected cases of isolated popliteal artery CAD when routine imaging suggests, but does not definitively prove, a communicating tract. By showing contrast passage from the knee joint into the adventitial cyst, this case supports the source-conduit-target model and illustrates how mechanism-directed imaging may complement standard vascular evaluation.

## Funding

No funding was received for this study.

## Disclosures

The authors declare no conflicts of interest.
